# The Redox Biology of Excitotoxic Processes: The NMDA Receptor, TOPA Quinone, and the Oxidative Liberation of Intracellular Zinc

**DOI:** 10.3389/fnins.2020.00778

**Published:** 2020-07-24

**Authors:** Elias Aizenman, Ralph H. Loring, Ian J. Reynolds, Paul A. Rosenberg

**Affiliations:** ^1^Department of Neurobiology, Pittsburgh Institute for Neurodegenerative Diseases, School of Medicine, University of Pittsburgh, Pittsburgh, PA, United States; ^2^Department of Pharmaceutical Sciences, School of Pharmacy, Northeastern University, Boston, MA, United States; ^3^Rewind Therapeutics, Leuven, Belgium; ^4^Program in Neuroscience, F.M. Kirby Neurobiology Center, Department of Neurology, Boston Children’s Hospital, Harvard Medical School, Boston, MA, United States

**Keywords:** excitotoxicity, redox, NMDA receptor, catecholamine, zinc, potassium channel

## Abstract

This special issue of Frontiers in Neuroscience-Neurodegeneration celebrates the 50th anniversary of John Olney’s seminal work introducing the concept of excitotoxicity as a mechanism for neuronal cell death. Since that time, fundamental research on the pathophysiological activation of glutamate receptors has played a central role in our understanding of excitotoxic cellular signaling pathways, leading to the discovery of many potential therapeutic targets in the treatment of acute or chronic/progressive neurodegenerative disorders. Importantly, excitotoxic signaling processes have been found repeatedly to be closely intertwined with oxidative cellular cascades. With this in mind, this review looks back at long-standing collaborative efforts by the authors linking cellular redox status and glutamate neurotoxicity, focusing first on the discovery of the redox modulatory site of the *N*-methyl-D-aspartate (NMDA) receptor, followed by the study of the oxidative conversion of 3,4-dihydroxyphenylalanine (DOPA) to the non-NMDA receptor agonist and neurotoxin 2,4,5-trihydroxyphenylalanine (TOPA) quinone. Finally, we summarize our work linking oxidative injury to the liberation of zinc from intracellular metal binding proteins, leading to the uncovering of a signaling mechanism connecting excitotoxicity with zinc-activated cell death-signaling cascades.

## Introduction

Reduction and oxidation reactions lie at the heart of critical biochemical processes indispensable for life. Indeed, there is no need to look much beyond oxidative phosphorylation to appreciate the essential nature of redox biology (Lehninger, 1945; Hill, 1951; Ernster and Lee, 1964). The aim of this review is to illustrate key redox processes as they relate to neuronal excitotoxicity that have been collaboratively explored by the authors over the last 30 years. In the first part of the review, we describe the experiments that led to the discovery of the redox modulatory site on the NMDA receptor (Aizenman et al., 1989). We then summarize the key observations that followed this finding, tightly linking the redox modulatory site to excitotoxic phenomena (Aizenman and Reynolds, 1992). In the second part of the review, we outline the studies we performed characterizing the oxidative conversion of the catecholamine precursor 3,4-dihydroxyphenylalanine (DOPA) to the kainate-like excitotoxin 2,4,5-trihydroxyphenylalanine (TOPA) quinone (Aizenman et al., 1990b; Rosenberg et al., 1991) culminating with the demonstration that TOPA quinone could be generated by catecholamine-containing cells, thereby introducing a novel mechanism of neurodegeneration in the study of endogenous neurotoxic processes in the brain (Newcomer et al., 1995b). We then transition to describe our work that first described the oxidative liberation of intracellular zinc in neurons (Aizenman et al., 2000). This process, which is closely associated with excitotoxic injury, results in a now-well characterized, complex signaling cascade that is an important component of neuronal cell death (Pal et al., 2004; Redman et al., 2009b; McCord and Aizenman, 2014; Shah and Aizenman, 2014). This review is intended as a retrospective of our own collaborative work rather than a comprehensive overview of the redox biology of excitotoxic phenomena. As such, we sincerely apologize in advance to all of our colleagues who have made significant contributions to this research topic but whom we have failed to cite.

## The NMDA Receptor Redox Modulatory Site

### Background

The NMDA appears to have been assembled in the course of evolution by tethering bacterial periplasmic amino acid-binding proteins onto an inside out potassium-like channel (Traynelis et al., 2010). This receptor is not only a gateway to long-term changes in synaptic function, but, strikingly, also a mediator of neuronal cell death (Hansen et al., 2017). Indeed, unregulated activation of this receptor is a major component of excitotoxicity (Foster et al., 1983; Choi et al., 1989), the aptly worded cell death process coined by the late John Olney, as a result of his work on glutamate neurotoxicity (Blood et al., 1969; Olney, 1969; Olney and Sharpe, 1969) following earlier observations by Lucas and Newhouse (1957). It is this potentially lethal risk of overactivity of NMDA receptors that may have contributed to the endowment of this ligand-gated channel with a profusion of modulatory domains. Zinc, protons, magnesium, glycine, polyamines, membrane surface tension, and even light comprise a partial list of known regulators of NMDA receptor function (Paoletti and Ascher, 1994; Leszkiewicz et al., 2000; Hansen et al., 2018). Our own work, first reported over 30 years ago, added another important component to this list, namely, redox-active agents (Aizenman et al., 1989). Through the years, many of these forms of NMDA receptor regulation, including the redox modulatory site (Jensen et al., 1994; Sanchez et al., 2000), have provided enticing, if ultimately frustrating, targets for the pharmacological alleviation of excitotoxic cell death. Below, we describe the studies that led to the discovery of the redox site as well as key observations from our laboratories that followed this finding.

### Redox Revealed

**FIGURE 1:**
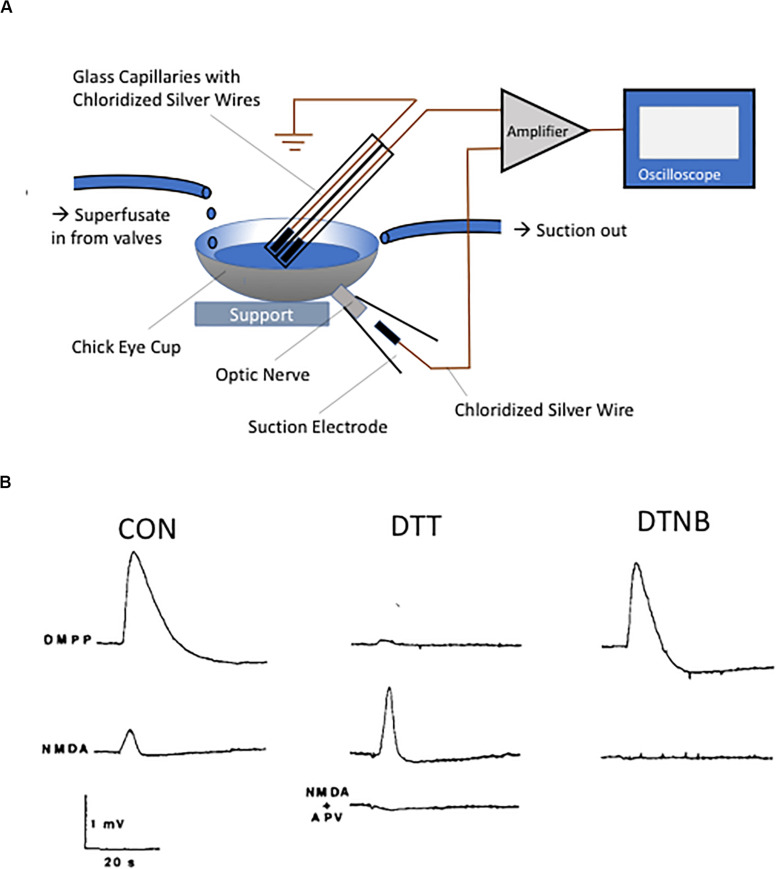
Discovery of the NMDA receptor redox modulatory site. **(A)** Diagram of the chick eyecup preparation. Eyecups were obtained from 2- to 5-day-old chicks and superfused with a buffered, oxygenated saline solution. The DC potential between a unipolar suction electrode placed on the cut optic nerve and a unipolar silver/silver chloride electrode placed inside the perfusion fluid was measured. **(B)** Responses obtained following application of either the nicotinic agonist DMPP (100 μM, top traces) or NMDA (100 μM, bottom traces) under control conditions or following sequential redox treatment with the 2 mM DTT (15 min) and 1 mM DTNB (5 min). Note the near absence of DMPP response following reduction with DTT, with restoration of the response after oxidation with DTNB. The NMDA-induced responses behaved in diametrically opposite direction, with a substantial potentiation of the response observed following DTT treatment. The NMDA receptor antagonist APV (100 μM, 10 min) completely blocked the NMDA response. Traces in **B** are reprinted from permission from Elsevier (Aizenman et al., 1989).

In the mid-1980s, two of the authors (EA and RL) began a collaborative effort aimed at biochemically isolating putative neuronal nicotinic receptors from the chick retina. Although at the time nicotinic receptors were widely appreciated to exist in the CNS, their now known complex molecular identity had just begun to be elucidated (Boulter et al., 1987; Goldman et al., 1987; Whiting et al., 1987). One of us (RL) had previously isolated a fraction of the venom from the banded krait *Bungarus multicinctus* (toxin F, later known as κ-bungarotoxin), which could act as a potential high affinity ligand for the immunoprecipitation of acetylcholine receptors from solubilized chick retinal membranes (Loring et al., 1984, 1986, 1989; Loring and Zigmond, 1988). Unfortunately, we found that virtually every detergent utilized in our studies promptly disrupted the binding site for the toxin, and therefore we turned our attention to isolating the receptor by affinity alkylation. This plan was based on earlier work by Arthur Karlin and colleagues describing the presence of redox-labile vicinal cysteine residues at the agonist binding site of the alpha subunit of the muscle nicotinic receptor (Karlin and Winnik, 1968; Karlin, 1969; Kao and Karlin, 1986). To confirm that the nicotinic receptors present in the chick retina contained redox-sensitive moieties similar to muscle receptors, we first monitored trans-retinal electrical activity following drug application to the retina, using the convenient hemisected chick eyeball as a perfusion chamber (Figure 1A). As anticipated, responses evoked by the selective nicotinic agonist 1,1-dimethyl-4-phenyl-piperazinium iodide (DMPP) were essentially abolished following a 2-min application of the reducing agent dithiothreitol (DTT), an effect reversible by a subsequent treatment with the thiol oxidizing agent 5,5’-dithio-*bis*-nitrobenzoic acid (DTNB) (Figure 1B). To control for potential deleterious actions of the thiol agents on the preparation, we utilized what we had thought would be a proper control, an excitatory agonist acting at a different receptor, likely not affected by redox agents. To our surprise, responses evoked by the glutamate receptor agonist *N*-methyl-D-aspartate (NMDA) were extremely sensitive to redox agents, and actually behaved in the opposite direction to those triggered by DMPP: DTT dramatically potentiated the electrical responses to NMDA, while DTNB nearly completely inhibited them (Figure 1B) (Aizenman et al., 1989).

The experiments that followed this finding essentially took three main directions of study. One path aimed to define the actions of redox agents on the intrinsic properties of the NMDA receptor while attempting to identify the molecular identity of the thiol reactive moieties within the protein. The second path was directed at identifying potentially endogenous modulators of the redox modulatory site. The third path aimed to establish the relevance of the site for NMDA receptor-mediated pathophysiological cellular processes. The results of these three research programs are briefly summarized below.

### Functional Properties of Redox Modulation of the NMDA Receptor

In addition to chick retina recordings, our initial study, conducted in the laboratories of Richard Zigmond and Stuart Lipton, also evaluated NMDA receptor-mediated whole cell currents in cultured rat cortical neurons under reducing and oxidizing conditions. We noted no changes in extracellular magnesium or zinc block, and no overt changes to the current–voltage relationship of the currents (Aizenman et al., 1989). The Reynolds and Aizenman laboratories quickly followed with a study that represented the first of a long string of collaborative redox studies between the two groups (Reynolds et al., 1990). We found that reducing and oxidizing conditions could effectively increase or decrease overall levels of glutamate, glycine, and spermidine-stimulated binding of the NMDA receptor channel blocker [^3^H]-MK801 to rat brain membranes, without any observable changes in the affinity of any of these agents. Moreover, redox agents did not affect the binding affinity of NMDA receptor competitive antagonists. We also observed dramatic enhancements in NMDA-stimulated intracellular calcium responses following DTT treatment. A detailed analysis of the redox properties of the NMDA receptor was then performed, utilizing whole-cell patch clamp recording in cortical neurons in tissue culture (Tang and Aizenman, 1993a). Here, we defined the time and concentration-dependence for reduction and oxidation of the receptor. Most informatively, we showed that the actions of redox agents on native NMDA receptors in neurons could be observed at the single channel level, primarily as a change in open channel frequency. Lastly, we were able to demonstrate that following reduction, the NMDA receptor could be “locked” in a potentiated state by utilizing the alkylating agent *n*-ethylmaleimide (Tang and Aizenman, 1993b). A follow-up study examined the voltage-dependence of reduction and oxidation, where we observed that reduction was much more effective at negative holding voltages in whole cell patch-clamped cortical neurons (Tang and Aizenman, 1993c). Interestingly, sequential reduction and oxidation at positive potentials led to long-lasting changes in the mean open time of receptor channels (Tang and Aizenman, 1993b).

### Subunit Localization of Redox Modulatory Sites of the NMDA Receptor

**FIGURE 2:**
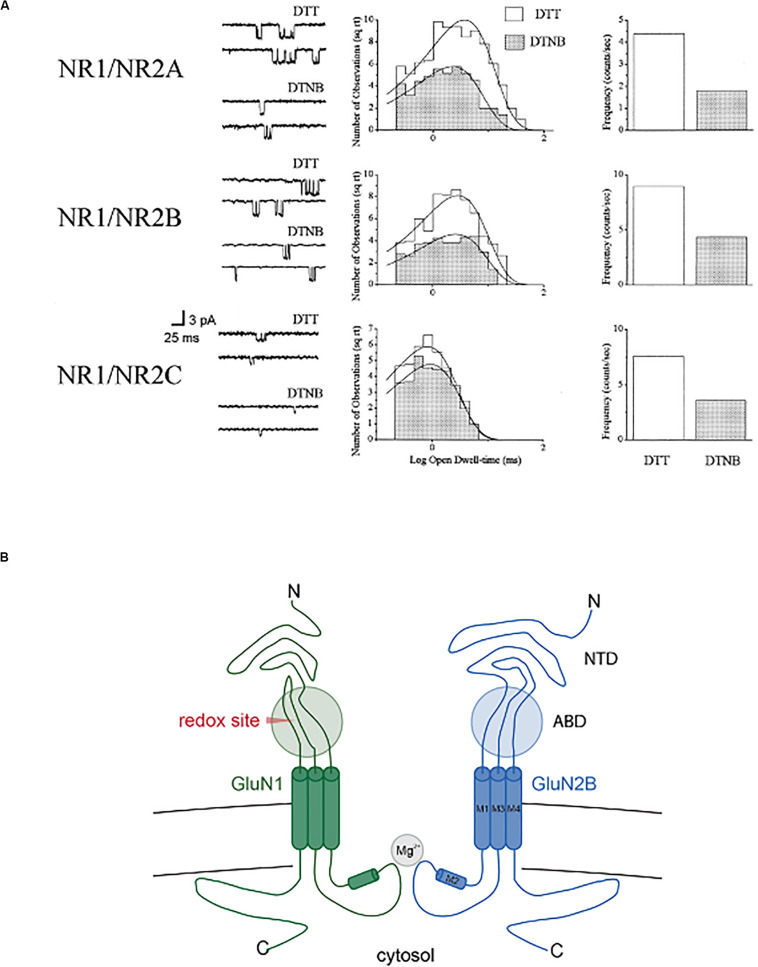
Redox modulation of single recombinant NMDA receptor channels. **(A)** NMDA (10 μM)-activated channels in outside out membrane patches (−60 mV) excised from CHO cell previously transfected with GluN1 in combination with either GluN2A, GluN2, or GluN2C (*n* = 4–9). Patches were exposed continuously to either 1 mM DTT or 0.1 mM DTNB. Open channel amplitudes were not affected by redox treatments (not shown). Open dwell-time histograms were fit by single exponential functions, with only redox-induced change observed in GluN2A-containing channels (DTT: 4.4 ± 0.4 ms; DTNB: 2.8 ± 0.2 ms; *p* < 0.0001, paired *t*-test; *n* = 9). Frequency of channel and probability of channel opening increased >2-fold for all subunit configurations. Copyright by the National Academy of Sciences of the United States of America; reprinted with permission (Brimecombe et al., 1997). **(B)** Simplified cross-section of a diheteromeric GluN2A/GluN2B receptor indicating the approximate location of the redox modulatory site (ABD, agonist binding domain; NTD, N-terminal domain).

A study by Sullivan et al. (1994) was the first to identify two extracellular cysteine residues within the GluN1 subunit that were critical for imparting redox sensitivity to heteromeric receptors assembled with GluN2B, GluN2C, or GluN2D subunits, but not GluN2A. This study suggested that GluN2A contained an additional redox-sensitive moiety, which our group later found to be sufficient for the modulation of GluN1/GluN2A receptors (Brimecombe et al., 1999), and selectively modified by cyanide, a known reducing agent (Arden et al., 1998). Work by Stuart Lipton and co-workers associated the GluN2A redox site, which was also extracellular, to the high sensitivity of this subunit to the metal zinc (Choi et al., 2001). We performed a series of single channel studies in recombinant NMDA receptors expressed in Chinese hamster ovary cells (Boeckman and Aizenman, 1994, 1996; Blanpied et al., 1997) to characterize in detail the roles the various receptor subunits played in redox modulation (Brimecombe et al., 1997). Reduction increases open channel frequency in all receptor combinations tested (GluN1/GluN2A, GluN1/GluN2B, GluN1/GluN2C), but only GluN2A-containing receptors had redox-dependent changes in open dwell-time (Figure 2). As a final note in this section, the two redox sensitive cysteine residues in GluN1 (Sullivan et al., 1994) were essential for imparting NMDA receptors with light sensitivity (Leszkiewicz et al., 2000, 2002), even in receptors containing the GluN2A subunit (Leszkiewicz and Aizenman, 2002). These studies revealed many opportunities for fine-tuning NMDA receptor responses by redox modulation, in a subunit-specific manner.

### Endogenous Modulators of the NMDA Redox Site

During our initial studies, we observed an intrinsic variability in the redox state of native NMDA receptors, such that in some cells the initial DTT potentiation was modest, with a subsequent, very pronounced inhibitory effect of DTNB; in most cells, however, the initial effect of the reductant was most pronounced (Aizenman et al., 1989). This suggested to us the possibility that endogenous thiol-reactive agents could be present in our preparations. Indeed, we soon discovered that redox-active oxygen-derived free radicals were highly effective at modifying NMDA receptor function (Aizenman et al., 1990a; Aizenman, 1995). The list of potential endogenous modulators of the redox site grew rapidly. Oxidized glutathione (Gilbert et al., 1991), the essential nutrient pyrroloquinoline quinone (PQQ) (Aizenman et al., 1992b, 1994; Scanlon et al., 1997), dihydrolipoic and lipoic acid (Tang and Aizenman, 1993a), as well as singlet oxygen (Eisenman et al., 2009), were all shown to interact with the redox site and influence NMDA receptor function.

**FIGURE 3:**
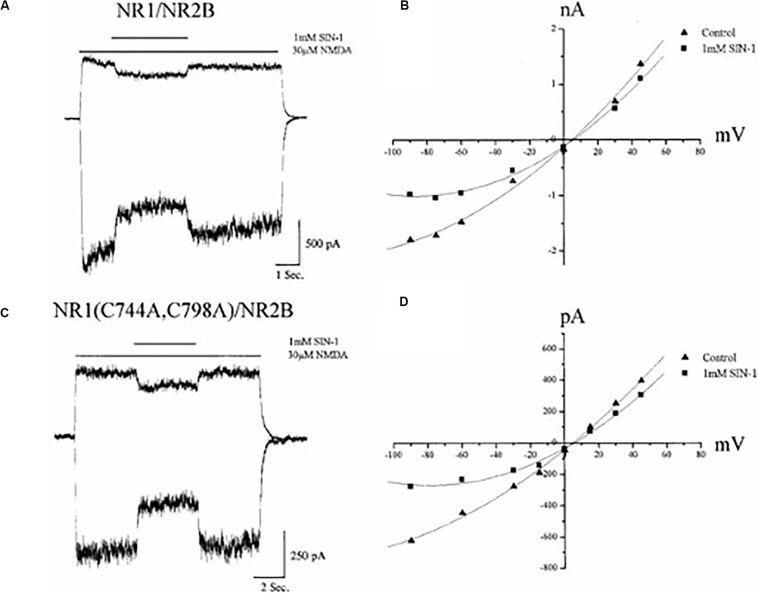
Nitric oxide inhibition of NMDA receptors does not require a functional redox site. **(A)** Whole–cell currents (−60, +30 mV) obtained in a CHO cell previously transfected with GluN1/GluN2B receptors during application of NMDA (30 μM) in the absence and presence of the nitric oxide donor SIN–1 (1 mM). Although SIN-1 produces superoxide in addition to nitric oxide, addition of superoxide dismutase and catalase do not influence the ability for this compound to inhibit NMDA-mediated responses (Fagni et al., 1995). Moreover, a variety of other NO donors have similar inhibitory effects (Fagni et al., 1995; Aizenman and Potthoff, 1999). **(B)** Current–voltage relationship of NMDA responses in the absence and presence of SIN-1 for the same cell shown in **A**. **(C)** Whole–cell currents (−60, +30 mV) from GluN1(C744A, C798A)/GluN2B receptors, which lack a functional redox site (Sullivan et al., 1994), during application of NMDA with or without SIN–1. **(D)** Current–voltage relationship of steady–state NMDA responses in the absence and presence of SIN-1 for the same cell shown in **C**. Reprinted with permission from John Wiley and Sons (Aizenman and Potthoff, 1999).

Our group found that nitric oxide (NO) could, under certain conditions, modify NMDA receptor physiological responses (Hoyt et al., 1992). Stuart Lipton and colleagues (Lei et al., 1992) reported that the effects of NO were, in fact, mediated via the modification of the NMDA receptor redox site. This conclusion was based on the observation that NO donors produced an attenuated inhibition in reduced and alkylated (“locked”) NMDA receptor-mediated calcium transients. In direct contrast to these findings, Joel Bockaert and colleagues noted that reduced and alkylated NMDA receptor-mediated whole-cell currents were still sensitive to NO donors, suggesting a lack of effect on the redox site (Fagni et al., 1995). In agreement with these findings, results from our group, utilizing recombinantly expressed wild-type and mutant GluN1 receptors lacking a redox site (Figure 3), established that the redox site was likely not the primary target of the reactive nitrogen species (Aizenman et al., 1998; Aizenman and Potthoff, 1999). Most importantly, later studies by John Garthwaite and colleagues indicated that physiological levels of NO did not influence NMDA receptor function to any extent (Hopper et al., 2004).

### Redox Modulation of NMDA Excitotoxicity

A number of thiol oxidizing agents acting at the redox site proved to be strongly neuroprotective against NMDA receptor-mediated excitotoxic injury, including the seleno-organic compound ebselen (Herin et al., 2001). Moreover, sub-lethal concentrations of oxygen-derived free radicals also limited NMDA toxicity, leading us to postulate that endogenously generated reactive species, acting via the redox modulatory site, may serve a neuroprotective role during tissue reperfusion following cerebral ischemia (Aizenman et al., 1990a). In contrast to oxidizing agents, reducing agents, such as DTT, potentiated NMDA excitotoxicity, an effect that could be completely blocked by antagonists of the NMDA receptor (Aizenman and Hartnett, 1992). Finally, our group provided evidence that changes in NMDA receptor redox sensitivity may be partly responsible for developmental changes in excitotoxic susceptibility in cortical neurons in tissue culture (Sinor et al., 1997). We noted that the NMDA receptor in immature neurons tended to prefer an oxidized basal state, while in mature neurons, the resting state of the receptor tended to rest in a more reduced state. Immature neurons, which are normally fairly resistant to NMDA toxicity, could thus be rendered sensitive to injury by the addition of a reducing agent (Sinor et al., 1997).

In the late 1980s, Paul Gallop (1927–1996) introduced the redox cycling quinone PQQ to two of us (PR and EA) while we were using an instrument in his well-equipped laboratory. Paul was a brilliant, engaging, kind, and generous Harvard biochemist. At the time we met him, PQQ seemed to occupy the forefront of his research efforts. This compound had been described as an essential nutrient, proposed to be a co-factor in many enzymatic redox reactions in a wide range of tissues (Gallop et al., 1989; Bishop et al., 1998). Paul suggested to us that PQQ may act as a strong modulator of the NMDA redox site, which was indeed the case (Aizenman et al., 1992b, 1994; Scanlon et al., 1997). Importantly, PQQ proved to be strongly neuroprotective, not only against excitotoxic injury in *in vitro* preparations (Aizenman et al., 1992b), but also in *in vivo* models of both stroke and epilepsy, as we showed in a series of studies in collaboration with Frances Jensen (Jensen et al., 1994; Sanchez et al., 2000). Unfortunately, during that time, the NMDA receptor as a drug target in human stroke had been losing favor due a series of failed clinical trials of NMDA receptor antagonists, and the emergence of data suggesting that some these agents produce a vacuolar degeneration (Olney et al., 1991; Fix et al., 1993; Ikonomidou and Turski, 2002; Hoyte et al., 2004). There remains the possibility that redox modulation of the NMDA receptor, as distinguished from receptor blockade, would offer an effective approach without the pathological consequences. Our work in this area was an exciting endeavor in which a chance discovery, followed by intensive mechanistic studies, led to a potentially translatable drug class. It is also noteworthy that work by other investigators over the years has provided important links between the NMDA redox site and a wide range of physiological and pathological processes in the brain, including NMDA receptor-mediated synaptic current long-term potentiation (Gozlan et al., 1995), NMDA receptor hypofunction in schizophrenia (Steullet et al., 2016), as well as synaptic dysfunction in aging and Alzheimer’s disease (Foster et al., 2017).

## TOPA Quinone, a Catechol-Derived Excitotoxin

### A Historical Perspective

From the late 1950s to the mid-1970s, Jeffrey Watkins and colleagues carried out a series of groundbreaking studies using amphibian and mammalian spinal cord preparations to test a large number of small molecules as potential excitatory and inhibitory neural substances. A powerful and now well know excitant resulting of this search was, of course, glutamate (Curtis et al., 1959). And, as they say, the rest is history (Watkins and Jane, 2006). But among some of the many compounds evaluated, these investigators noted that both L-3,4-dihydroxyphenylalanine (L-DOPA) and its hydroxylated derivative L-2,4,5-trihydroxyphenylalanine (6-hydroxydopa, or TOPA), could also elicit excitatory activity in their preparations (Biscoe et al., 1976). These observations were of special interest, given the known inherent instability and neurotoxicity of oxidant-prone catechols such as dopamine (Graham, 1978; Rosenberg, 1988) and concerns about the possibility that L-DOPA or levodopa, the most important treatment for Parkinson’s disease, might itself be the cause of neurodegeneration in populations of dopaminergic and non-dopaminergic neurons (Bonnet et al., 1987; Fahn, 1996; Agid et al., 1998).

**FIGURE 4:**
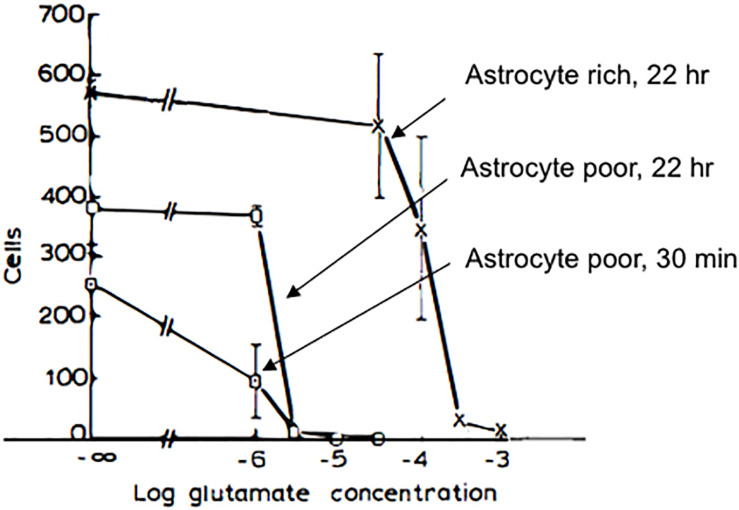
Pharmacology of glutamate toxicity in astrocyte-poor and in astrocyte-rich cultures. Astrocyte-poor and astrocyte-rich cultures were compared for their sensitivity to glutamate toxicity. Cultures were exposed to glutamate at selected concentrations in MEM either for 22 h (cross, astrocyte-rich; open box, astrocyte-poor) or for 30 min (filled box, astrocyte-poor). In the latter case, after glutamate exposure, medium was replaced with MEM, and cultures were returned to incubator for 16.5 h. Experiments were terminated by replacing media with 2.5% glutaraldehyde in physiological saline. Neurons were identified as phase bright cells with typical morphology (see text). For astrocyte-poor cultures, 30-min incubation, cultures were used at 20 days *in vitro*, and 5 fields/coverslip were counted. Data shown are from one experiment. For 22 h incubation, astrocyte-rich and astrocyte-poor cultures at 4 weeks *in vitro* and derived from the same plating were tested for sensitivity to glutamate toxicity in the same experiment, and 10 fields/coverslip were counted. In astrocyte-poor cultures, virtually no neurons survived in 3 μM glutamate, with either an overnight or a 30 min incubation. In astrocyte-rich cultures, a significant loss of neurons was seen only at 300 μM glutamate. Modified and reprinted with permission from Elsevier (Rosenberg and Aizenman, 1989).

At approximately the same time that PAR was studying the toxic potential of catecholamines to CNS neurons in culture (Rosenberg, 1988), we began our collaboration with a study that revealed the extreme sensitivity of CNS neurons to excitotoxicity (Rosenberg and Aizenman, 1989) (Figure 4). Remarkably, when neurons were grown in culture without astrocytes, L-glutamate was found to have an LD50 (concentration at which 50% of neurons are killed following treatment) in the micromolar concentration range, a degree of vulnerability to the toxic effects of glutamate that had not been previously appreciated (Rosenberg et al., 1992; Lipton and Rosenberg, 1994). It was recognized that there may be multiple mechanisms responsible for the influence of astrocytes on the potency of glutamate as an excitotoxin. We established that NMDA receptor-mediated currents were unaffected by the presence or absence of astrocytes (Rosenberg and Aizenman, 1989). In addition, NMDA receptor agonists that were not substrates for glutamate transporters, including NMDA itself, had virtually identical excitotoxic potencies regardless of the culture type (Rosenberg et al., 1992). This work, along with observations by Garthwaite et al. (1992), demonstrated that glutamate uptake was a critical determinant of neuronal survival in the mammalian CNS. Moreover, our work established that the pharmacology of NMDA receptor-mediated toxicity, specifically regarding the potency of agonists, was similar to the pharmacology of the NMDA receptor characterized by measuring NMDA mediated physiological responses and binding studies (Rosenberg et al., 1992). This conclusion had been previously missed in previous work on excitotoxicity that did not take into account the effects of glutamate uptake on the interaction of glutamate agonists with their receptors, as had been anticipated by Garthwaite (1985). In 1992, the three major glutamate transporters of the forebrain were cloned, including EAAC1 (Kanai and Hediger, 1992), GLAST (Storck et al., 1992), and GLT-1 (Pines et al., 1992), which represent approximately 1% of total protein in the forebrain (Lehre and Danbolt, 1998). Although GLT-1 for over a decade after its discovery was assumed to be expressed exclusively in astrocytes (Rimmele and Rosenberg, 2016), it was ultimately shown to be the primary glutamate transporter expressed in axon terminals (Chen et al., 2004; Petr et al., 2015) where it serves an important metabolic function in providing glutamate as a substrate for synaptic mitochondria (McNair et al., 2020). Recent work has strongly implicated GLT-1 in the pathogenesis of Alzheimer’s disease (Sharma et al., 2019; Zott et al., 2019) emphasizing the importance of understanding the fine regulation of the concentrations of glutamate and other glutamate agonists in and around synapses.

**FIGURE 5:**
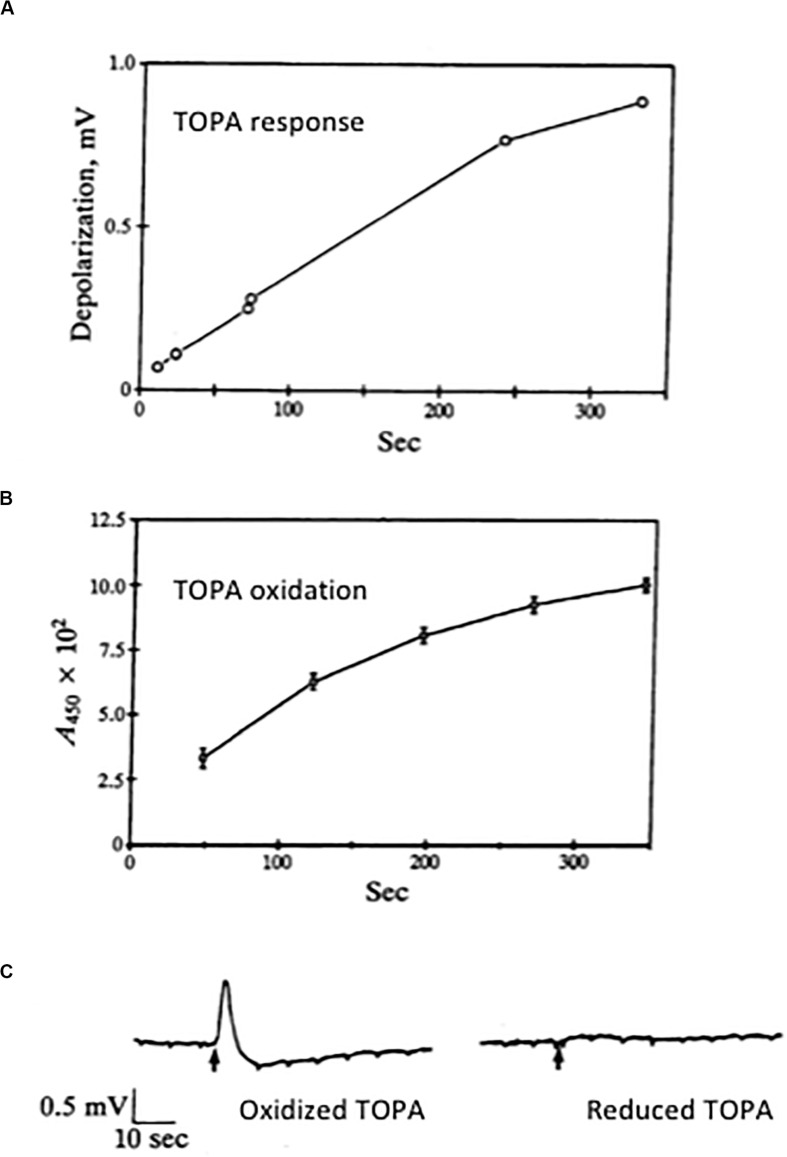
Excitatory actions of an oxidation product of TOPA. **(A)** A fresh solution of 300 μM TOPA was prepared from a 10 mM stock prepared in 1 mM HCl. At selected times, aliquots of this solution were perfused onto a chick eye cup preparation; peak responses were plotted. **(B)** A similar TOPA solution was prepared and at selected times its absorbance at 450 nm was measured. All results represent the mean ± SD of three experiments. Note the increase response obtained as the solution oxidizes to a colored product. **(C)** Excitatory responses in the chick eye-cup preparation to applications of 300 μM TOPA in the absence (oxidized) and presence (reduced) of 3 mM DTT. Oxidized TOPA is a non-NMDA agonist (Aizenman et al., 1990b). Copyright by the National Academy of Sciences of the United States of America; modified and reprinted with permission (Rosenberg et al., 1991).

We were thus open to the possibility that even low-level production of the glutamate receptor agonist TOPA by oxidation of L-DOPA *in situ* might be a significant cause of excitotoxic neurodegeneration. In collaboration with RHL, we were able to demonstrate that, in our hands, freshly prepared DOPA itself was not an excitant (Newcomer et al., 1995a) while TOPA was an efficacious excitatory agonist acting at non-NMDA receptors (Aizenman et al., 1990b). Of note, while we were enduring a lengthy and tortuous review process of our manuscript, Olney et al. (1990) reported excitatory actions of TOPA. In a subsequent, more comprehensive study, we showed that, in fact, an oxidation product of TOPA, putatively TOPA quinone, and not TOPA itself, was the excitatory substance (Rosenberg et al., 1991). As TOPA oxidized into a red-colored quinone product, the ability of the solution to elicit excitatory responses greatly increased (Figure 5). We also found that the oxidized form of TOPA, which was spectrophotometrically distinct from dopachrome, was strongly excitotoxic to cultured rat cultured neurons via non-NMDA receptor activation (Rosenberg et al., 1991). Maintaining TOPA in a reduced state also limited its ability to excite or kill neurons (Rosenberg et al., 1991; Aizenman et al., 1992a). In collaboration with Paul Gallop, we attempted to identify the active species using mass spectrometry but poor volatilization of the amino acid and inability to form stable adducts limited our progress. We thus turned our attention to high-performance liquid chromatography (HPLC) to address this issue.

### TOPA Quinone

**FIGURE 6:**
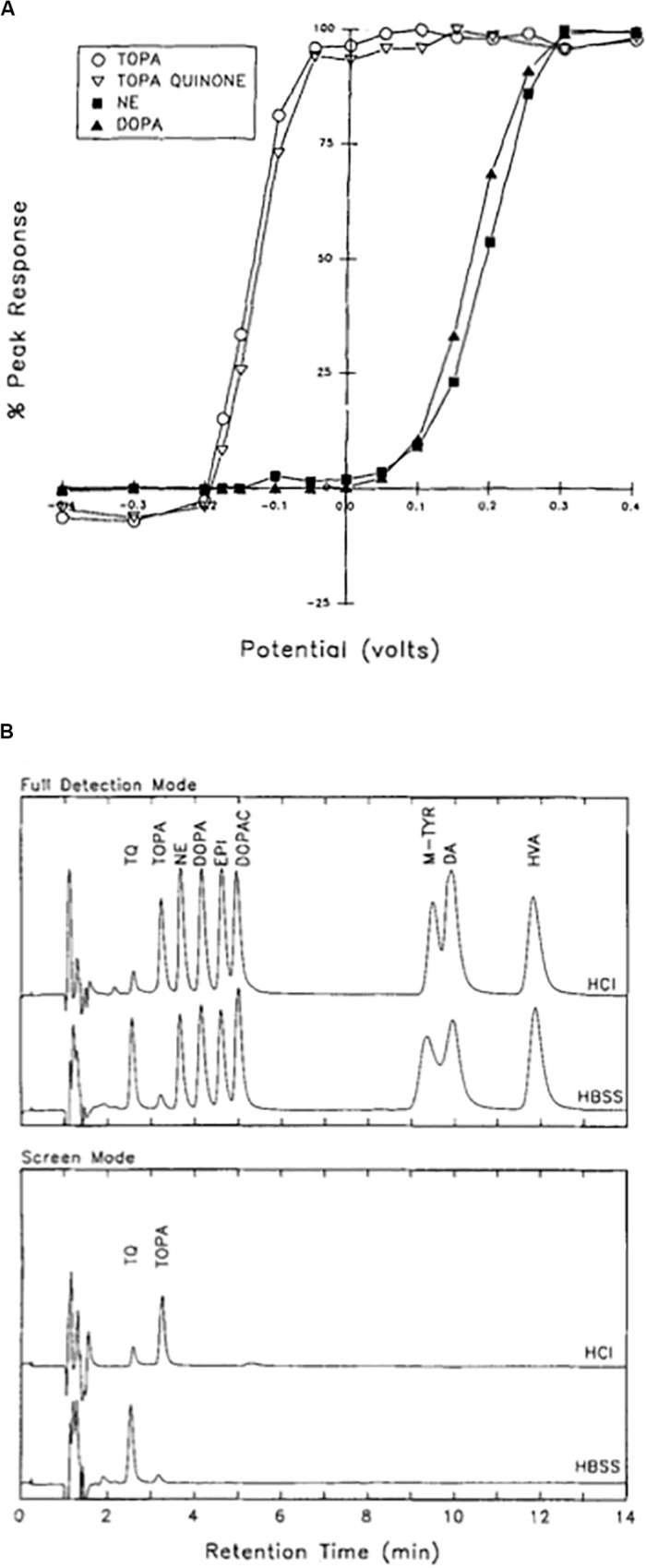
Isolation of TOPA quinone in physiological solutions. **(A)** Current–voltage curves for TOPA, TOPA quinone, norepinephrine (NE), and DOPA measured at the electrochemical detector following HPLC separation. Values are normalized to the current values obtained at 0.4 V. Note the left-sided shift in the oxidation profile for TOPA and TOPA quinone when compared to NE and DOPA. **(B)** TOPA and TOPA quinone can be selectively detected by excluding all other closely related compounds. In the full detection mode (top), the detection electrode was at 0.4 V and the reference electrode at −0.4 V. In the screen mode, the detection electrode was at −0.075 mV, with the reference electrode remained at −0.4 V. Samples were injected at either neutral pH in HBSS or in 0.01 M HCl. TQ, TOPA quinone; NE, norepinephrine; EPI, epinephrine; DOPAC, 3,4-dihydroxyphenylacetic acid; M-TYR, meta-tyrosine; DA, dopamine. Note how TOPA and TOPA quinone are the only compounds detected in the screen mode, as all other catechol-derived compounds have failed to oxidize at the selected detection voltage. Reprinted with permission from John Wiley and Sons (Newcomer et al., 1993).

**FIGURE 7:**
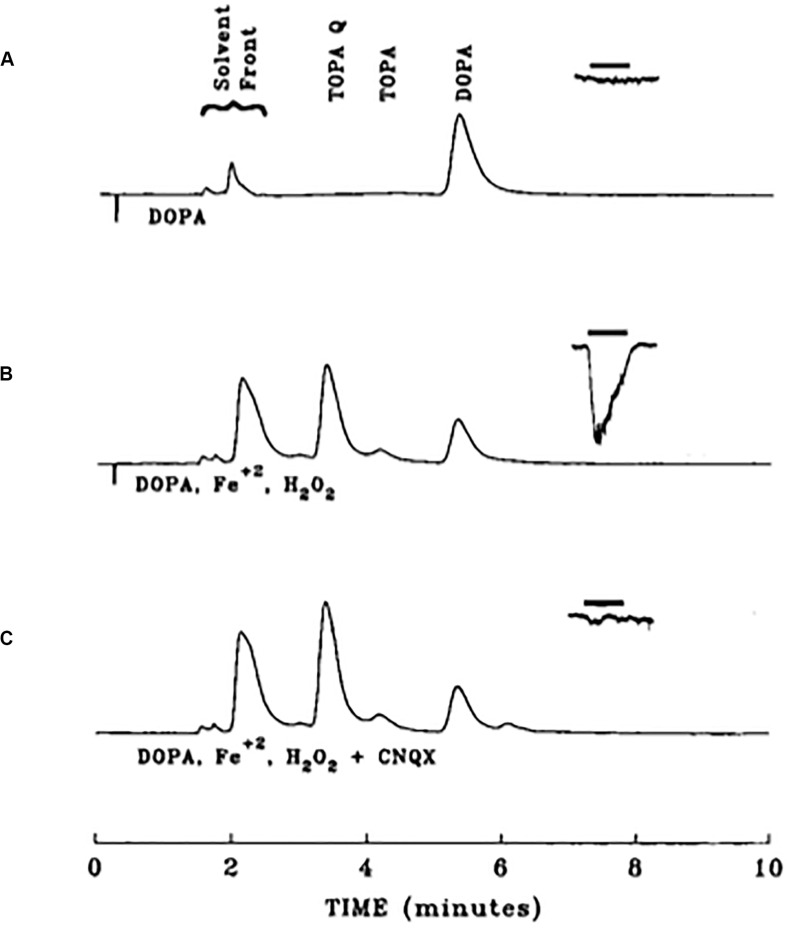
Iron-mediated conversion of DOPA to an excitotoxin. HPLC chromatograms from three different 1 mM DOPA-containing solutions, overlaid with resultant whole cell currents produced by application of those substances onto rat cortical neurons in tissue culture (−90 mV). **(A)** Chromatogram of a DOPA solution freshly prepared in HBSS and allowed to sit for 30 min. The same solution was applied for 3 s to a cortical neurons, eliciting no response. **(B)** Chromatogram of a DOPA solution allowed to incubate for 30 min in the presence of 1 mM ferrous iron and 10 mM hydrogen peroxide. This solution was applied to a cortical neuron, eliciting a response, attributable to a production of approximately 25 μM TOPA quinone. **(C)** Same conditions as in **B**, but in the presence of 10 μM of the non-NMDA receptor antagonist CNQX. Note the lack of response elicited by this solution. Reprinted with permission from John Wiley and Sons (Newcomer et al., 1995a).

In the mid-1990s, we performed a series of detailed analytical studies using reverse-phase HPLC coupled to a dual electrode coulometric detector to analyze both DOPA and TOPA-containing solutions under a variety of conditions (Newcomer et al., 1993). One of the most striking features of our analysis was the revelation that both TOPA and its autoxidation product TOPA quinone had a much more negative redox potential (−150 mV), when compared to nearly all other catecholamines tested (+200 mV) (Figure 6A). This feature allowed us to set the electrochemical detector at a potential where the chromatogram response would be maximal for TOPA and TOPA quinone, without virtually any interference from any other catechol-derived compound (Figure 6B). We also found that TOPA quinone was the dominant species at physiological pH, and that the conversion of TOPA to TOPA quinone was reversible upon acidification. Importantly, TOPA solutions allowed to oxidize for up to 4 h produced only one major product, with spectrophotometrically distinct profile from all other catecholamine oxidation products, such as dopachrome and dopa quinone (Graham, 1978; Rosenberg et al., 1991). Finally, we showed that DOPA-containing solution could also generate TOPA quinone, a process that we later demonstrated was facilitated by the presence of iron and hydrogen peroxide (Newcomer et al., 1995a) (Figure 7).

### Oxidative Production of TOPA Quinone by a Catecholaminergic Cell

What was missing as evidence to support our initial hypothesis was a demonstration that TOPA could be produced *in situ*. In the last study from our group on this subject, we showed that PC12 cells, a catecholaminergic cell line derived from rat pheochromocytoma (Greene and Tischler, 1976), were able to generate TOPA quinone under both basal and potassium-stimulated conditions (Newcomer et al., 1995b). Indeed, we were able to detect significant concentrations of both TOPA and TOPA quinone in the extracellular fluid of the cell cultures, a process facilitated by the addition of an inhibitor of DOPA decarboxylase. As no TOPA compounds were detected when glutathione was included in the incubating solution, we concluded that the oxidative conversion of DOPA to TOPA quinone occurred extracellularly. These results suggested that catecholaminergic cells in the brain could, under certain conditions, generate TOPA quinone and lead to excitotoxic injury, a notion reinforced by the fact that a prior lesion of the substantia nigra can protect striatal neurons from ischemia, an insult thought to cause neurodegeneration by excitotoxic processes (Globus et al., 1987). The excitatory and possible toxic consequences of TOPA quinone production continued to be discussed in the literature, many years after our work concluded (Guatteo et al., 2013; Kostrzewa, 2016). Finally, as a final note of interest demonstrating its endogenous production, TOPA quinone was identified as the quinone cofactor of several oxidases, some of which were previously thought to contain PQQ at its active site (Janes et al., 1990; Dove et al., 1996; Dooley, 1999).

## Oxidative Liberation of Intracellular Zinc

### Zinc Neurotoxicity

**FIGURE 8:**
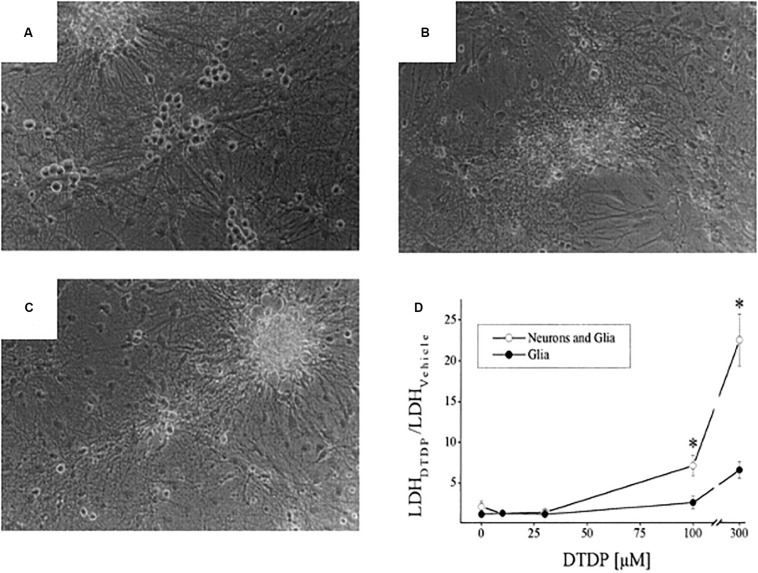
Neurotoxicity of DTDP, a thiol oxidant species. Cortical cultures containing a mix of neurons and glia were exposed to either: vehicle **(A)**, DTDP (100 μM, 10 min; **B**), or NMDA (100 μM, 30 min; **C**) and photographed 24 h later. Neurons are phase bright cells sitting atop of a glial cell layer. Note the similar pattern of neurotoxicity triggered by DTDP and NMDA. **(D)** DTDP concentration-cell death curves for cultures containing a mixture of neurons and glia or only glia. DTDP exposure was for 10 min and lactate dehydrogenase (LDH) assays, an index of cell death (Aras et al., 2008) were performed 18–20 h later. Note that DTDP kills neurons preferentially over glia (*p < 0.05). Modified from earlier work (Aizenman et al., 2000) and reprinted with permission from John Wiley and Sons.

**FIGURE 9:**
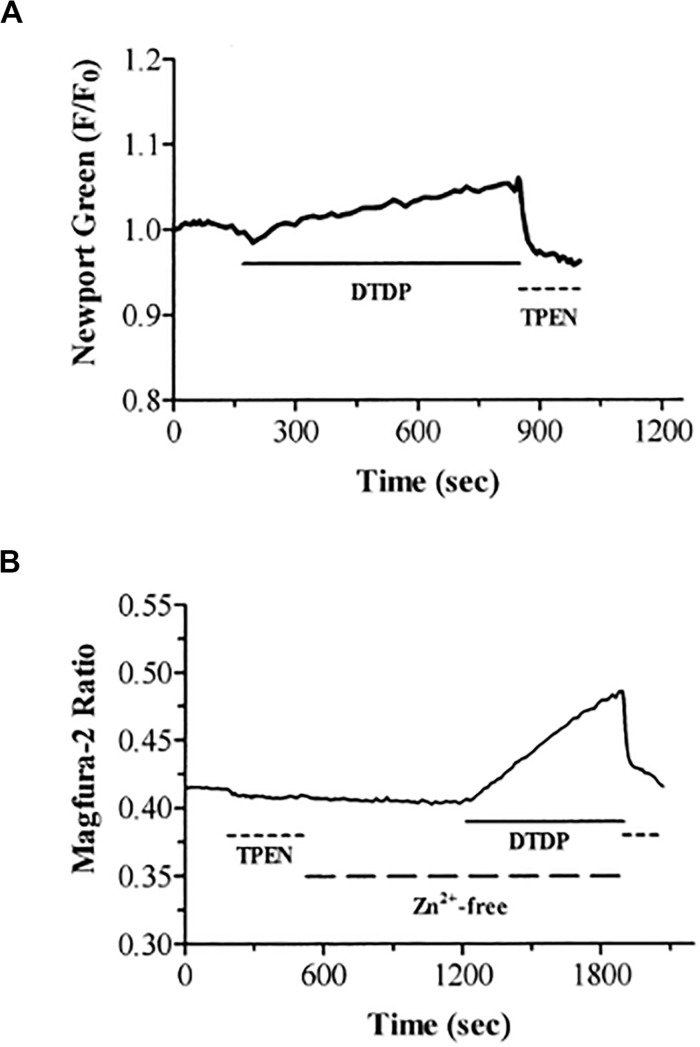
DTDP causes increases in intracellular zinc in neurons. **(A)** Neurons loaded with the zinc-specific fluorophore Newport Green were exposed to DTDP (100 μM). DTDP responses were reversed by treatment with TPEN (25 μM). **(B)** DTDP releases zinc from intracellular stores. Neurons loaded with magfura–2 were exposed to TPEN (25 μM) and subsequently washed with zinc–free buffer (large dashed line). After 10 min, DTDP (100 μM) was added to the zinc-free perfusate for an additional 10 min. The experiment was terminated with application of TPEN (25 μM). Previous studies in our laboratories have demonstrated the usefulness of the fluorescent indicators magfura–5, magfura–2, fura–2, and fluo–3 for measurement of intracellular zinc under conditions in which the intracellular free calcium concentration does not change (Sensi et al., 1997; Cheng and Reynolds, 1998). Reprinted with permission from John Wiley and Sons (Aizenman et al., 2000).

In the mid to late 1980s, Jae-young Koh, Dennis Choi, and colleagues showed that a brief exposure to zinc was sufficient to lethally injure cortical neurons in tissue culture and the field of zinc mediated neurodegeneration was born (Yokoyama et al., 1986; Choi et al., 1988). A few years later, Koh and colleagues observed that, following injury, zinc accumulated in vulnerable neurons in the absence of synaptic, or vesicular zinc, suggesting that the metal may have an intracellular source that, in and of itself, would be sufficient to trigger cell death (Lee et al., 2000). Indeed, that same year, two of the authors (EA and IR) provided unequivocal evidence that oxidative liberation of intracellular zinc from metal-binding proteins could induce a neuronal cell death signaling cascade that resembled, in many ways, excitotoxicity, especially in its preference for neurons over glia (Figure 8) (Aizenman et al., 2000). In those initial studies, we utilized the cell-permeant thiol oxidizing agent 2,2′-dithiodipyridine (DTDP) and were able to demonstrate release of zinc from intracellular stores (Figure 9). Many reviews have appeared summarizing our work and that of others on this subject (Pal et al., 2004; Aras and Aizenman, 2011; Sensi et al., 2011; McCord and Aizenman, 2014), including a very recently published book chapter (Aizenman, 2019). As such, we focus here on the specific connections that exist between excitotoxic processes and the liberation of intracellular, mobile zinc.

### Intracellular Zinc Liberation

Zinc can be released from intracellular metal-binding proteins, primarily metallothionein (MT), via redox-mediated processes (Maret, 2019). In fact, MT, in spite of having a very high affinity for zinc (Kd = 1 × 10^–14^ M at physiological pH), can readily release the metal upon mild oxidative conditions (−365 mV redox potential) (Maret and Vallee, 1998). Wolfgang Maret, Bert Vallee, and colleagues utilized DTDP to oxidize zinc-coordinating residues within MT and release its bound zinc (Maret et al., 1997; Jacob et al., 1998; Jiang et al., 1998; Maret and Vallee, 1998). This work alerted us to the possibility that DTDP was lethally injuring neurons by inducing zinc release, rather than by a triggering a calcium-dependent process, which would have been the more obvious choice given the prominent role assigned to calcium in excitotoxic neurodegeneration (Aizenman et al., 2000). In fact, we had initially hypothesized that the lethal actions of the oxidizing agent were being mediated by release of calcium from the endoplasmic reticulum following oxidation of the ryanodine receptor, as reported to occur in cardiac myocytes by Prabhu and Salama (1990). We were initially led astray by the promiscuous divalent cation binding ability of most calcium indicators (Grynkiewicz et al., 1985), but ultimately demonstrated the critical role of mobilization of free zinc (Aizenman et al., 2000). We now know that MT, especially MT3, the primary isoform in neurons (Hidalgo et al., 2001; Vasak and Meloni, 2017), represents a critical source of intracellular zinc in injured neurons (Lee et al., 2003), can serve both as a source and a buffer of intracellular zinc in astrocytes (Malaiyandi et al., 2004), and is a key and highly regulated component of cell death signaling pathways triggered by the metal (Sensi et al., 2003; Aras et al., 2009a; Medvedeva et al., 2017). However, with an estimated 2800 zinc binding proteins in the human proteome (Andreini et al., 2006), there is clearly the possibility of a multitude of proteins contributing to an intracellular zinc response.

### Linking Excitotoxic Stimuli, Intracellular Zinc Release, and Neuronal Dysfunction

**FIGURE 10:**
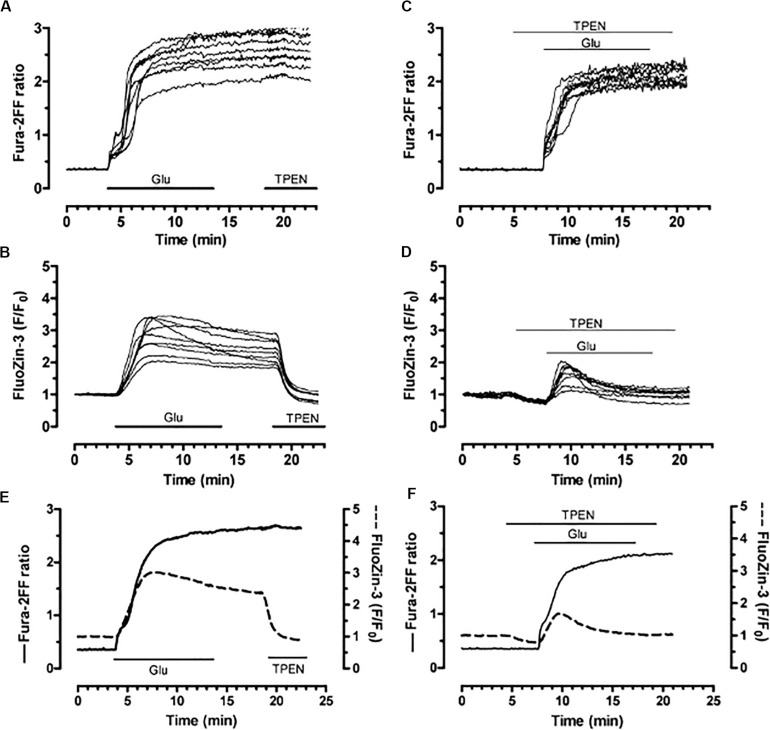
Simultaneous detection of glutamate–induced intracellular calcium and zinc in neurons. Neurons loaded with both fura–2FF (fluorescent calcium indicator) and FluoZin–3 (fluorescent zinc indicator) were exposed to glutamate (100 μM; in the presence of 10 μM glycine; 10 min). **(A,B)** Fura–2FF and FluoZin–3 traces following sequential glutamate and TPEN (5 μM) treatments. **(C,D)** Fura–2FF and FluoZin–3 signals from neurons stimulated with glutamate and co–treated with TPEN (2 μM) before, during, and after the glutamate stimulus. **(E)** Mean traces from a group of neurons treated with glutamate. **(F)** Mean traces from a group of neurons treated with glutamate and TPEN. Fura–2FF is indicated by solid trace and corresponds to left *y*–axis; FluoZin–3 is indicated by dashed trace and corresponds to right *y*–axis. Reprinted with permission from John Wiley and Sons (Dineley et al., 2008).

Intracellular zinc release in neurons can be induced by a variety of stimuli in addition to DTDP. These include peroxynitrite (Zhang et al., 2004, 2006; Knoch et al., 2008), ischemic injury (Aras et al., 2009b), and, importantly, glutamate (Aras et al., 2009a). As glutamate can also generate the production of reactive oxygen species via NMDA receptor activation (Reynolds and Hastings, 1995), by simultaneously measuring calcium and zinc transients in neurons (Figure 10), the Reynolds laboratory provided unequivocal evidence that NMDA receptor stimulation leads to a calcium-dependent production of free radicals, both cytoplasmic and mitochondrial in origin, which, in turn, led to the liberation of intracellular zinc (Dineley et al., 2008). This finding, and subsequent work by us and other groups (Granzotto and Sensi, 2015; Yeh et al., 2017, 2019), provided a critical link between excitotoxic processes and intracellular liberated zinc-dependent cell injurious signaling cascades. Zinc translocation from presynaptic, zinc containing terminals to postsynaptic cells via calcium-permeable channels (Sensi et al., 1999a, b) can, under certain conditions, also contribute to the deleterious actions of the metal (Medvedeva et al., 2017). Of note, increases in intracellular zinc can lead to subsequent additional calcium-dependent processes that significantly contribute to neuronal death-inducing cellular pathways (Vander Jagt et al., 2009; McCord and Aizenman, 2013).

In addition to the generation of reactive oxygen species, dysregulated intracellular zinc has other well-known effects on mitochondrial function, including alterations in mitochondrial membrane potential (Sensi et al., 2000, 2003; He and Aizenman, 2010), altered trafficking (Malaiyandi et al., 2005), and mitochondrial channel opening with the subsequent release of pro-apoptotic factors (Jiang et al., 2001; Bonanni et al., 2006). However, activation of apoptotic signals requires the loss of cytoplasmic potassium, providing a requisite, optimum environment for protease and nuclease activation (Bortner et al., 1997; Hughes et al., 1997; Hughes and Cidlowski, 1999; Montague et al., 1999). Our group thus began an investigation to evaluate whether cellular potassium efflux was also required for intracellular zinc-mediated cell death, and if so, what cell signaling components were activated to accomplish this result.

### A Zinc-Potassium Continuum in Neuronal Cell Death

**FIGURE 11:**
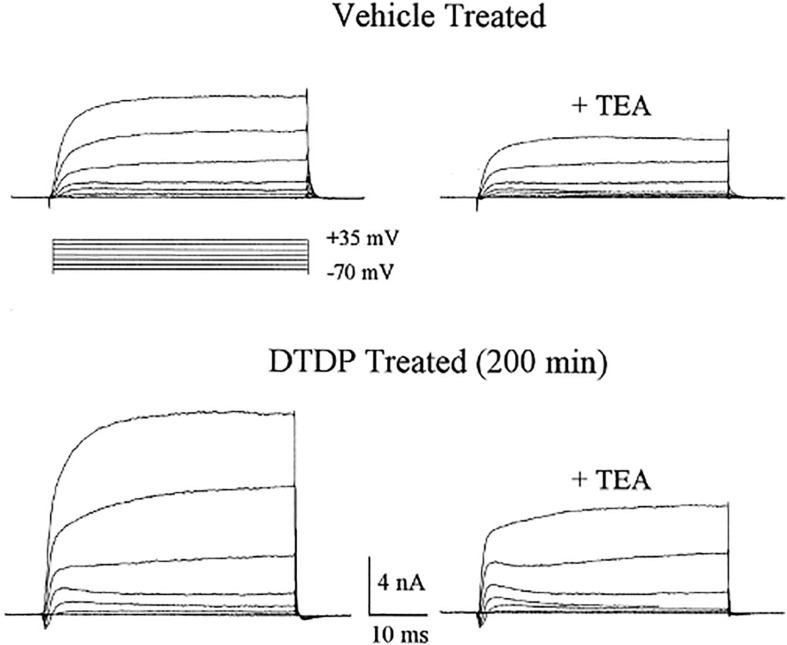
DTDP-induced enhancement of voltage-gated potassium currents in cortical neurons. Whole-cell potassium currents obtained in two separate cortical neurons ∼3 h after a 10 min exposure to either vehicle or 100 μM DTDP. Potassium currents were evoked by a series of voltage steps to +35 mV from a holding potential of −70 mV. Note that currents are substantially larger in the DTDP-treated cell, when compared with the vehicle-treated neuron. TEA (10 mm)blocked ∼50% of the currents in both cases. Modified and reprinted with permission from the Society for Neuroscience (McLaughlin et al., 2001).

Neurons exposed to DTDP were monitored for the enhancement of potassium currents as had been reported by Yu et al. (1997) in serum-deprived and staurosporine treated cells destined for apoptotic cell death. We observed a remarkable enhancement in delayed-rectifier, tetraethylammonium (TEA)-sensitive potassium currents approximately 3 h after a brief DTDP exposure (McLaughlin et al., 2001) (Figure 11). The potassium current surge could be blocked by zinc chelation and p38 MAPK inhibitors, but not by caspase inhibitors. However, all of these three treatments, in addition to TEA, could block DTDP toxicity, suggesting that zinc and p38 were upstream of the enhanced potassium currents, later identified as being mediated by the delayed rectifier potassium channel Kv2.1 (Pal et al., 2003). Additional studies revealed that the apoptotic potassium current surge results from a zinc-activated phosphorylation of Kv2.1 by both Src and p38, which in turn, induces a syntaxin-dependent *de novo* insertion of large number of Kv2.1 channels into the plasma membrane (Pal et al., 2006; Redman et al., 2007, 2009a; McCord et al., 2014). The characterization of this cell death-enabling pathway led to the generation of a new generation of neuroprotective compounds for the treatment of neurodegenerative disorders in which excitotoxicity has been proposed to play a prominent role, including cerebral ischemia-reperfusion injury (Yeh et al., 2017, 2019). The efficacy of these agents suggests either that the zinc mobilizing pathway that we have delineated and the excitotoxic pathway are both independently active in ischemic injury, or that these two pathways are intimately linked. Finally, it must be noted that zinc-triggered cell death pathways are not always apoptotic in nature, nor dependent on potassium efflux. For example, we observed that the neurotoxic action of the biocide methylisothiazolinone is also mediated through intracellular zinc liberation, in this case resulting in ERK activation and caspase-independent cell death (Du et al., 2002). In addition, Lee and Koh (2010) have reported that oxidative liberation of intracellular zinc can also lead to lysosomal dysfunction and autophagy in neurons and astrocytes.

## Concluding Remarks

In this article, we have looked back over 30 years of collaborative work on many aspects of redox processes important in neurodegeneration. Writing this review has provided the occasion for assessing the impact our research has had not only on the field of excitotoxicity, but also on neuroscience in general. Importantly, it has stimulated us to think about where the field is now, and what there might be left to contribute. Clearly, what is missing from the field of excitotoxicity in general is the translation of an enormous body of work into useful clinical drugs to halt or limit neuronal cell death. This has been, admittedly, a very difficult problem, to which large amounts of financial resources and numerous careers have been devoted. Our own recent work points to new therapeutic avenues to pursue, targeting Kv2.1-mediated potassium efflux in cell death processes (Yeh et al., 2017, 2019; Schulien et al., 2020). Although ameliorating excitotoxic injury and clinical disability has been very challenging, a better appreciation of critical mechanisms can only improve the potential to succeed in addressing this important unmet medical need. Regardless of any potential future developments, however, we feel our past and current interactions, and the work that has resulted from our many collaborative efforts, have been highly stimulating, intellectually rewarding, but, most importantly, incredibly fun. Here is looking at the next 50 years of excitotoxicity research!

## Author Contributions

All authors contributed to the writing of this review article.

## Conflict of Interest

IR is employed by company Rewind Therapeutics. The remaining authors declare that the research was conducted in the absence of any commercial or financial relationships that could be construed as a potential conflict of interest.
